# Rehabilitation success and related costs following stroke in a regional hospital: a retrospective analysis based on the Australian National Subacute and Non-Acute Patient (AN-SNAP) classification

**DOI:** 10.1186/s12913-024-12090-w

**Published:** 2025-01-23

**Authors:** Fan He, George Mnatzaganian, Michael Njovu, David Rutherford, Tara Alexander, Irene Blackberry

**Affiliations:** 1 John Richards Centre for Rural Ageing Research, La Trobe Rural Health School, College of Science, Health and Engineering, La Trobe University, Edwards Road, Flora Hill, VIC 3550, Victoria, Australia; 2 Melbourne School of Population and Global Health, University of Melbourne, Melbourne, VIC Australia; 3 Care Economy Research Institute, La Trobe University, Albury-Wodonga, Victoria, Australia; 4 Rural Department of Community Health, La Trobe Rural Health School, College of Science, Health and Engineering, La Trobe University, Bendigo, VIC Australia; 5 Rehabilitation Medicine Department, Albury Wodonga Health, Wodonga, VIC Australia; 6 School of Clinical Medicine, Albury Campus, University of New South Wales, Albury, NSW Australia; 7 Division of Medicine, Albury Wodonga Health, Albury, NSW Australia; 8 Australasian Rehabilitation Outcomes Centre, University of Wollongong, Wollongong, NSW Australia

**Keywords:** Stroke, Rehabilitation, Success, AN-SNAP, Payment, Regional, Australia, Functional Independence Measure

## Abstract

**Background:**

Evidence is limited on the factors influencing successful stroke rehabilitation in regional contexts. Additionally, the relationship between rehabilitation costs following acute stroke, based on Australian National Subacute and Non-Acute Patient (AN-SNAP) casemix classification, and rehabilitation success remains unclear.

**Objective:**

This retrospective cohort study investigated the factors contributing to improved functional outcomes following stroke rehabilitation in an Australian regional hospital, also evaluating the respective average daily and total payments.

**Methods:**

Stroke patients’ admission records, during 2010–2020, were linked with rehabilitation registry data. Rehabilitation success was defined as relative functional gain (RFG) ≥ 0.5 and Functional Independence Measure (FIM) efficiency ≥ 1. Multivariate mixed effects logistical regressions modelled the sociodemographic and medical (i.e., comorbidities and stroke type) predictors of rehabilitation success, while logarithms of average daily and total rehabilitation payments were modelled using robust regressions.

**Results:**

Of 582 included patients, 315 (54.1%) achieved RFG ≥ 0.5 and 258 (52.2%) achieved FIM efficiency ≥ 1. A longer delay in starting rehabilitation was associated with a lower likelihood of achieving RFG success [Odds Ratio (OR): 0.85, 95% confidence interval (CI): 0.78–0.93, *P* < 0.001] and FIM efficiency success (OR: 0.89, 95% CI: 0.82–0.97, *P* = 0.010). A higher FIM score at admission was associated with decreased odds of FIM efficiency success (OR: 0.35, 95% CI: 0.20–0.60, *P* < 0.001). The average daily and total rehabilitation payments for inpatients were $AU1,255 (median) [interquartile range (IQR): 1,040, 1,771] and $AU28,363 (median) (IQR: 18,822, 41,815), respectively. FIM efficiency success was positively associated with the average daily payment (Beta: 0.25, 95% CI: 0.20–0.30, *P* < 0.001), but negatively correlated with the total payment (Beta: -0.18, 95% CI: -0.24–0.13, *P* < 0.001). No significant associations were found between RFG success and these payments.

**Conclusion:**

This study identifies key factors affecting stroke rehabilitation outcomes in a regional Australian setting. Delays in starting rehabilitation were linked to lower success rates, underscoring the importance of timely intervention. While higher average daily costs were associated with better FIM efficiency, total costs did not correlate with relative functional gains. These findings may inform rehabilitation practices and may influence future funding strategies for rehabilitation services.

**Supplementary Information:**

The online version contains supplementary material available at 10.1186/s12913-024-12090-w.

## Introduction

Stroke is a leading cause of global mortality and disability [1], ranking as the third highest cause of death in Australia [2]. Various factors contribute to disabilities in stroke survivors, including the stroke event itself, musculoskeletal problems, sensory impairments, and other complications [3]. Rehabilitation is crucial in the sub-acute phase of stroke treatment, as it considerably improves both immediate and long-term functional abilities [4], regardless of age, stroke type, severity, or whether it is a first or recurring event [5–7]. Stroke rehabilitation strives to help patients achieve maximum independence – physically, psychologically, socially, and financially – despite lasting impairments [8]. However, current literature primarily defines successful recovery or rehabilitation in terms of functional improvements.

While rehabilitation success is frequently tied to functional recovery levels, definitions of successful recovery can vary greatly. This includes measures of neurological impairment, functional disabilities, and quality of life, adding complexity to quantifying stroke recovery [9]. Numerous scales are utilised to assess stroke recovery, including the Fugl-Meyer Assessment [10], National Institutes of Health Stroke Scale [11], Physical Function Index [12], Modified Rankin Scale [13], Barthel Index (BI) [14], and Functional Independence Measure (FIM) [15]. While all measures are reliable and valid [9, 15], FIM and BI are the most commonly used [16],with FIM being more comprehensive as it includes cognitive items and more response categories [16, 17]. Studies have defined successful rehabilitation with specific FIM measurements, such as a 10% change in score [18], relative functional gain (RFG) of 0.5 or higher [19], and FIM efficiency of one or more [19]. RFG quantifies functional gains relative to the maximum possible gains, while FIM efficiency measures the speed of regaining functions [20]. The preference of FIM assessments over alternative measures facilitates the standardisation of successful rehabilitation definitions. In Australia, both urban and rural rehabilitation facilities use FIM to assess stroke patients upon admission and discharge. Nevertheless, individuals residing in regional areas are frequently underrepresented in studies, resulting in limited evidence on factors linked to successful rehabilitation in those settings.

Modern rehabilitation requires a multidisciplinary care team, [21] making it crucial to assess the effectiveness and funding of this resource-intensive intervention [16]. In Australia, an activity-based funding system has been established to compensate for the number and diversity of treated patients [22]. This reform aims to increase transparency in funding usage and promote efficient resource allocation in hospitals [23]. To implement this funding model, Australia developed both the national efficient price [24] and the casemix classification for categorising different patients [25]. Adjustments for geographical differences, private insurance, Indigenous status, costly treatments, sentinel events, hospital-acquired complications, and unplanned readmissions were also introduced to improve care quality [25]. However, the current activity-based funding model, based on cost analysis and casemix classifications, does not directly align with patient recovery outcomes. There exists a gap in understanding how this funding relates to the functional recovery of stroke patients. Furthermore, it is unclear whether the current rehabilitation funding model will provide financial incentives for hospitals to help stroke patients in achieving successful rehabilitation outcomes.

The objective of this study was to investigate the factors associated with success in RFG and FIM efficiency among stroke survivors. Additionally, this study evaluated whether these successes could potentially result in economic benefits for the hospital in terms of payments based on the current activity-based funding system.

## Methods

### Data sources and ethics

This ten-year retrospective cohort gathered de-identified data on patient demographics, diagnoses, comorbidities, and admissions from the routinely collected Electronic Medical Records system at the Albury Wodonga Health (AWH). The Australasian Rehabilitation Outcomes Centre (AROC) supplied the rehabilitation records of all patients included in the study, obtained through a probabilistic data linkage performed by AROC.

Ethics clearance was obtained from the Human Research Ethics Committees of AWH (HREC/73611/AWHEC-2021–256466), La Trobe University (HREC73611), and Australian Institute of Health and Welfare (EO2021/5/1311).

### Population description

AWH is a healthcare centre in regional Australia which oversees a significant catchment area including 10 local government areas. Situated on the border between the two most populous states (New South Wales and Victoria) [26], AWH serves a diverse population of around 280,000 individuals living in various communities in Southern New South Wales and Northeast Victoria [27]. AWH comprises two campuses, Albury Hospital and Wodonga Hospital, offering a range of services including acute, sub-acute, emergency, and mental health care [28].

This study included adult patients (≥ 18 years) who were consecutively admitted to AWH between January 2010 and December 2020 with a first or recurrent stroke (haemorrhagic, ischemic, or unspecified) and had registered inpatient rehabilitation records (from AWH or another hospital) in AROC. Patients included in this study had at least two FIM assessments recorded, at the time of admission to rehabilitation and just prior to discharge. Patients were excluded if their primary diagnosis was not a stroke or if they died during their index admissions. Patients with linked rehabilitation that commenced a year after being discharged from the initial stroke admission were also excluded, because this episode of rehabilitation was not considered relevant to the initial onset of stroke.

The International Statistical Classification of Diseases and Related Health Problems (ICD 10th Revision) was used to determine the primary diagnosis of stroke. Specifically, haemorrhagic stroke was identified by I60 (nontraumatic subarachnoid haemorrhage) and I61 (nontraumatic intracerebral haemorrhage), ischaemic stroke by I63 (cerebral infarction), and stroke of unspecified type by I64 (stroke which is not specified as haemorrhage or infarction).

### Outcomes

The primary outcomes of this study encompassed success in Function Independence Measure (FIM) Relative Functional Gain (RFG) [20], and success in FIM efficiency [20]. Total rehabilitation payment and average daily rehabilitation payment were analysed.

#### Success in Functional Independence Measure (FIM) Relative Functional Gain (RFG)

The FIM is composed of 18 items that evaluate a patient’s motor and cognitive abilities, with each item being rated on a seven-point ordinal scale [29]. The motor component of FIM assesses a patient’s performance in self-care, transfers, ambulation or wheelchair use, stairs use, and bowel and bladder management. Meanwhile, the cognitive component evaluates a patient’s expression, understanding, problem-solving, memory, and social interaction. The total FIM score can vary between 18 and 126, with higher scores indicating better functional performance. Clinicians who assess the FIM are trained and credentialed in its use to ensure consistent scoring throughout Australia.

The FIM RFG assessed the progress a patient made after rehabilitation, in comparison to the maximum potential improvement based on the patient’s functional level upon admission [20]. As the FIM RFG approaches one, the patient is approaching their maximum potential improvement from the initial functional level upon admission. If there is no change in a patient’s FIM score from admission to discharge, the RFG would be zero, indicating a lack of improvement in functional independence.

The Relative Functional Gain can be calculated using the following set of formulas [30]:

- If the FIM score at discharge is greater than that at admission, RFG = (Discharge FIM—Admission FIM) / (126—Admission FIM)
- If the FIM score at discharge is less than that at admission, RFG = (Discharge FIM—Admission FIM) / Admission FIM
- If the FIM score at discharge is equal to the score at admission, RFG = 0

RFG success was defined as FIM RFG ≥ 0.5 [19], indicating that the patient had gained 50% of the maximum potential gain.

#### Success in FIM efficiency

The daily rate of patients’ functional improvement during their stay in rehabilitation was evaluated using FIM efficiency [20], which was calculated by dividing the absolute difference between the FIM scores at admission and discharge by the number of days in rehabilitation [30]. FIM efficiency success was defined as FIM efficiency ≥ 1 [19], suggesting that the patient could, on average, improve by at least 1 FIM point per day.

#### Total rehabilitation payment

The Australian National Subacute and Non-Acute Patient (AN-SNAP) casemix classification V5, modified by the Independent Health and Aged Care Pricing Authority (IHACPA), is used for activity-based funding [31]. Rehabilitation is one of the five subacute care types included in the AN-SNAP classification. The total rehabilitation payment for each patient was determined using AN-SNAP V5 classes. This was combined with the price weights for admitted subacute and non-acute patients, as per the AN-SNAP V5 classes for the 2023–2024 period.

The AN-SNAP V5 classes for patients were determined using the weighted FIM motor scores, FIM cognition scores at the time of rehabilitation admission, and the patient’s age [31]. A detailed method for calculating the weighted FIM motor scores can be found in *Appendix H (Impairment-specific FIM™ Motor item weights) of* AN-SNAP V5 manual [31].

The total rehabilitation payment for each patient’s rehabilitation episode was calculated using the price weights for the 2023–2024 period, in conjunction with the associated categories of length of stay in rehabilitation. The categories, as defined by the Independent Health and Aged Care Pricing Authority (IHACPA) in Australia, encompass low outliers, inliers, and high outliers.

The boundaries for these categories are determined through the following steps [24, 32]:

1. The national average length of stay (ALOS) for each AN-SNAP V5 class is calculated.
2. The boundary between the low outlier and inlier is determined by dividing the ALOS by 1.5 and rounding down to the nearest whole number.
3. The boundary between the high outlier and inlier is determined by multiplying the ALOS by 1.5 and rounding to the closest whole number.

As a result, the cut-off for the number of days constituting low outlier, inlier, and high outlier lengths of stay in rehabilitation varies across each AN-SNAP V5 class.

Different price weights were assigned to various lengths of stay categories, namely low outlier, inlier, and higher outliers, which also varied across different AN-SNAP V5 classes. Patients falling into the low outlier category had their payment determined by multiplying the national efficient price of $AU6,032 for 2023–2024 period [25] by the price weight corresponding to each day and length of stay. For patients categorised as inliers, their payment was calculated by multiplying the national efficient price of $AU 6,032 [25] by the price weight assigned to the entire rehabilitation episode. The inlier category has the greatest price weight, and the total payment remains unchanged within this category. Patients classified as higher outliers had their payment calculated by combining the amount for inlier payment with the payment for extra stays beyond the standard length of stay. The latter was calculated using national efficient price multiplied by the price weight for extra stays and number of days spent in extra stays.

The total payment for rehabilitation, as determined by the existing funding system, is a crude amount. This is primarily due to its failure to incorporate adjustments for unaccounted variations beyond the scope of the AN-SNAP classification. These adjustments are hindered by incomplete data items necessary for these adjustments, such as costly treatments, preventable readmissions, sentinel events (preventable adverse patient safety incidents with severe consequences), and hospital-acquired complications [25].

#### Average daily rehabilitation payment

The average daily payment for rehabilitation was obtained by dividing the total payment by the length of stay in rehabilitation.

### Covariates

This study’s covariates were patient’s age on index stroke admission, sex, Indigenous status, the remoteness of their residence, country of birth, comorbidities, type of stroke, stroke severity, delay in commencing rehabilitation, functional outcomes upon discharge from rehabilitation, and details on the index admission for acute stroke. Stroke severity were assessed using the National Institutes of Health Stroke Scale (NIHSS), with higher scores denoting poorer neurological function [33]. Patient’s ability to walk independently at the time of acute admission has been established as a validated measure of stroke severity [34] and was also included as covariate. The patient’s socio-economic status was determined using the Index of Relative Socio-economic Advantage and Disadvantage (IRSAD), which was based on their residential postcode [35]. The remoteness of a patient’s residence was classified using the Modified Monash Model (MMM) [36]. Data were collected on pre-stroke care needs (including paid and unpaid carers), pre-stroke service utilisation (paid and unpaid), and employment status prior stroke.

The covariates obtained from the index admission for acute stroke encompassed the type of admission ward, which included the stroke unit, intensive care unit (ICU), and coronary care unit (CCU). Documentation also included whether the admission occurred on a weekday, or a weekend/holiday. The time of admission was categorised as either daytime (from 8 am to 4 pm) or night-time (from 4 pm to 8 am). Additionally, the number of days from the onset of the stroke to the initiation of rehabilitation was also considered.

Comorbidities assessed as impacting participation in rehabilitation were identified from the linked AROC records, which included conditions such as respiratory disease, cardiac disease, dementia/delirium, drug and alcohol abuse, hearing/visual impairment, mental health issues, morbid obesity, diabetes mellitus, chronic pain, arthritis/osteoarthritis/osteoporosis, renal failure, and cancer. The number of comorbidities upon admission for stroke was estimated using all reported diagnoses.

Patients were categorized according to their admission FIM motor scores as follows: those with scores between 51 and 91 were classified as having high motor performance, those with scores between 36 and 50 were considered to have moderate motor performance, those with scores between 19 and 35 were identified as having low motor performance, and those with scores between 13 and 18 were characterized as having very low motor performance [20]. When the commencement of a patient’s rehabilitation exceeded 24 h from the point clinicians declared them fit for rehabilitation, it was noted as a delay in the initiation of rehabilitation. The specific number of days of delay in starting the rehabilitation was documented. Additionally, any complications that arose during rehabilitation and prevented patients from engaging in the planned intensity of the rehabilitation program were recorded. Reported complications included urinary tract infections, delirium, fractures secondary to falls, pressure ulcers, wound infections, venous thromboembolisms, chest infections, significant electrolyte imbalances, falls, faecal impactions, and infections with COVID-19.

### Statistical analysis

Chi-squared test or Fisher’s exact test (for categorical variables), and Wilcoxon rank sum test (continuous variables) were employed to compare the characteristics of patients who did or did not attain RFG success and FIM efficiency success. These statistical tests were also used to compare the characteristics of included and excluded patients. The RFG success and FIM efficiency success were modelled using mixed effects logistical regressions, with FIM motor group serving as the sole grouping variable to account for variations in the baseline functional performance upon rehabilitation admission. Upon evaluating the potential collinearity through pairwise Spearman tests, variables associated with study outcome (with p ≤ 0.1) in univariate models were incorporated into multivariate models. These multivariate mixed effects logistical regression model also adjusted for age, sex, type of stroke, treatment in a stroke unit or ICU or CCU, and number of comorbidities. The association between various factors and RFG success or FIM efficiency success was examined by plotting the odds ratios of fixed effects and intercepts of random effects from multivariate mixed effects logistic regression. This was done while considering variations in different FIM motor groups upon rehabilitation admission.

Patients were compared based on whether they achieved RFG success or not, considering factors such as average daily rehabilitation payment, total rehabilitation payment, and rehabilitation funding model payment categories (inlier and hi/low outlier) as determined by the length of stay within each AN-SNAP class. A similar comparative analysis was conducted between patients who attained FIM efficiency success and those who did not. In multivariate robust regressions that modelled the logarithm of the average daily rehabilitation payment and the logarithm of the total rehabilitation payment, age, sex, type of stroke, and the number of comorbidities were always adjusted for. After assessing the potential collinearity using pairwise Spearman tests, variables that were associated with the study measure with *p* value ≤ 0.1 at the univariate level were also included. To normalise the distribution of calculated payments, a logarithmic transformation was applied to these values for use in the regression model. This transformation resulted in a more symmetric distribution with a stabilised variance. In this semi-logarithmic model, the coefficient of the independent variable signifies the percentage change in the average daily rehabilitation payment or the total rehabilitation payment corresponding to a one-unit change in the independent variable.

Statistical significance was set at a *p*-value of ≤ 0.05 (two-sided). The analyses were performed using R.

## Results

This research found 607 stroke survivors from AWH who also had matching records in AROC. Patients were excluded if their rehabilitation started a year after their initial stroke admission (*n* = 23) or their FIM score at discharge was missing (*n* = 2). No age, stroke type, nor stroke severity differences were detected between patients included and excluded in this study (Table S1). However, the excluded patients were more likely to have been male than female (Table S1). Of the remaining 582 patients, a further 88 were excluded in the FIM efficiency success analysis as their rehabilitation stay length was not recorded.

As shown in Table 1, a higher proportion of younger patients achieved RFG success compared with older patients. The proportion of male patients was also larger among those achieving RFG success. Patients with successful RFG had fewer comorbidities, lesser days delayed in initiating rehabilitation, shorter duration from onset of stroke to rehabilitation, and a higher FIM total score upon admission. They were less likely to have been retirees, and less likely to have had experienced complications during rehabilitation. They also had a lower prevalence of existing conditions of cardiac disease, dementia, arthritis or osteoarthritis or osteoporosis. People living in private residences were less likely to have had a carer or to have received living assistive services prior to the stroke onset.

**Table 1:** Patient’s characteristics by success in Relative Functional Gain and Functional Independence Measure efficiency

	**Relative functional gain success** **(> = 0.5)**			**Functional Independence Measure efficiency success** **(> = 1)**		
**Variable**	**No**, *N* = 267^1^	**Yes**, *N* = 315^1^	***p*-value**^2^	**No**, *N* = 236^1^	**Yes**, *N* = 258^1^	***p*-value**^2^
**Age group (years)**^**a**^			< 0.001			0.377
Under 75	78 (29.2%)	179 (56.8%)		104 (44.1%)	122 (47.3%)	
75–84	110 (41.2%)	89 (28.3%)		74 (31.4%)	86 (33.3%)	
85 or more	79 (29.6%)	47 (14.9%)		58 (24.6%)	50 (19.4%)	
**Sex**^**a**^			< 0.001			0.088
Male	124 (46.4%)	199 (63.2%)		121 (51.3%)	153 (59.3%)	
Female	143 (53.6%)	116 (36.8%)		115 (48.7%)	105 (40.7%)	
**Indigenous status**^**ac**^			0.421			1.000
Indigenous	–-	–-		–-	–-	
Not Indigenous	–-	–-		–-	–-	
**Modified Monash Model remoteness**^**a**^			0.472			0.272
Metropolitan area/regional centre	166 (62.6%)	187 (59.4%)		151 (64.5%)	153 (59.3%)	
Rural area	99 (37.4%)	128 (40.6%)		83 (35.5%)	105 (40.7%)	
**Country of birth**^**a**^			0.416			0.894
Australia	123 (46.1%)	128 (40.6%)		107 (45.3%)	112 (43.4%)	
Foreign countries	14 (5.2%)	19 (6.0%)		16 (6.8%)	17 (6.6%)	
Unknown	130 (48.7%)	168 (53.3%)		113 (47.9%)	129 (50.0%)	
**Type of stroke**^**a**^			0.642			0.059
Stroke of unspecified type	19 (7.1%)	22 (7.0%)		14 (5.9%)	23 (8.9%)	
Ischaemic stroke	220 (82.4%)	252 (80.0%)		201 (85.2%)	198 (76.7%)	
Haemorrhagic stroke	28 (10.5%)	41 (13.0%)		21 (8.9%)	37 (14.3%)	
**Treated in a stroke unit or ICU or CCU**^**a**^			0.558			0.982
No	175 (65.5%)	198 (62.9%)		147 (62.3%)	162 (62.8%)	
Yes	92 (34.5%)	117 (37.1%)		89 (37.7%)	96 (37.2%)	
**Number of comorbidities**^**a**^	2 (2.0, 3.0)	2 (1.0, 2.0)	0.008	2 (2.0, 2.3)	2 (1.0, 2.8)	0.432
**Admitted on weekday or non weekday**^**a**^			0.382			0.581
Business day	210 (78.7%)	237 (75.2%)		177 (75.0%)	200 (77.5%)	
Weekend/Holiday	57 (21.3%)	78 (24.8%)		59 (25.0%)	58 (22.5%)	
**Days delayed in starting rehabilitation**^**b**^	1 (0.0, 3.0)	1 (0.0, 2.0)	< 0.001	1 (0.0, 3.0)	1 (0.0, 2.0)	0.015
**From onset of stroke to rehabilitation (days)**	11 (7.0, 17.0)	8 (5.0, 11.0)	< 0.001	9 (6.0, 15.0)	8 (5.0, 11.0)	< 0.001
**FIM total score**^**b**^	61 (38.0, 87.0)	84 (63.0, 99.0)	< 0.001	75 (46.0, 98.3)	82 (63.0, 94.8)	0.031
**Admission FIM motor group**^**b**^			< 0.001			< 0.001
Very low motor	58 (21.7%)	9 (2.9%)		37 (15.7%)	7 (2.7%)	
Low motor	52 (19.5%)	48 (15.2%)		49 (20.8%)	27 (10.5%)	
Moderate motor	45 (16.9%)	51 (16.2%)		27 (11.4%)	52 (20.2%)	
High motor	112 (41.9%)	207 (65.7%)		123 (52.1%)	172 (66.7%)	
**Rehabilitation services provider**^**b**^			0.299			0.094
Public	248 (92.9%)	284 (90.2%)		219 (92.8%)	227 (88.0%)	
Private	19 (7.1%)	31 (9.8%)		17 (7.2%)	31 (12.0%)	
**Insurance status**^**b**^			0.106			0.871
Public patient	135 (50.6%)	157 (49.8%)		119 (50.4%)	124 (48.1%)	
Private health insurance	91 (34.1%)	126 (40.0%)		89 (37.7%)	102 (39.5%)	
Others	41 (15.4%)	32 (10.2%)		28 (11.9%)	32 (12.4%)	
**Existing comorbidity: Cardiac disease**^**b**^	66 (24.7%)	52 (16.5%)	0.019	56 (23.7%)	43 (16.7%)	0.065
**Existing comorbidity: Respiratory disease**^**b**^	21 (7.9%)	21 (6.7%)	0.692	19 (8.1%)	16 (6.2%)	0.532
**Existing comorbidity: Drug and Alcohol abuse**^**bc**^	11 (4.1%)	6 (1.9%)	0.140	–-	–-	0.581
**Existing comorbidity: Dementia/delirium**^**bc**^	–-	–-	0.001	6 (2.5%)	14 (5.4%)	0.163
**Existing comorbidity: Mental health problem**^**b**^	22 (8.2%)	22 (7.0%)	0.679	15 (6.4%)	23 (8.9%)	0.370
**Existing comorbidity: Hearing/visual impairment**^**b**^	16 (6.0%)	10 (3.2%)	0.150	11 (4.7%)	12 (4.7%)	1.000
**Existing comorbidity: Diabetes mellites**^**b**^	36 (13.5%)	34 (10.8%)	0.386	27 (11.4%)	25 (9.7%)	0.627
**Existing comorbidity: Morbid obesity**^**bc**^	8 (3.0%)	6 (1.9%)	0.427	–-	–-	0.008
**Existing comorbidity: Arthritis/osteoarthritis/osteoporosis**^**b**^	45 (16.9%)	30 (9.5%)	0.012	32 (13.6%)	31 (12.0%)	0.705
**Existing comorbidity: Chronic pain**^**bc**^	10 (3.7%)	6 (1.9%)	0.272	9 (3.8%)	–-	0.325
**Existing comorbidity: Cancer**^**b**^	13 (4.9%)	10 (3.2%)	0.405	10 (4.2%)	8 (3.1%)	0.665
**Existing comorbidity: Renal failure**^**bc**^	–-	–-	1.000	–-	–-	0.743
**Existing comorbidity: Other**^**b**^	57 (21.3%)	37 (11.7%)	0.002	34 (14.4%)	40 (15.5%)	0.830
**Need for a carer prior to stroke (among those living in a private residence)**			0.004			0.446
Having carer	59 (24.8%)	43 (13.7%)		145 (61.4%)	160 (62.0%)	
No carer	142 (59.7%)	215 (68.3%)		39 (16.5%)	51 (19.8%)	
Unknown	37 (15.5%)	57 (18.1%)		52 (22.0%)	47 (18.2%)	
**Need for any services prior to stroke (among those living in a private residence)**			0.004			0.351
No	144 (53.9%)	212 (67.3%)		137 (58.1%)	166 (64.3%)	
Yes	66 (24.7%)	57 (18.1%)		52 (22.0%)	47 (18.2%)	
Unknown	57 (21.3%)	46 (14.6%)		47 (19.9%)	45 (17.4%)	
**Employment status prior to stroke**			< 0.001			0.751
Retired	196 (73.4%)	174 (55.2%)		152 (64.4%)	158 (61.2%)	
Employed	20 (7.5%)	72 (22.9%)		39 (16.5%)	48 (18.6%)	
Not employed	51 (19.1%)	69 (21.9%)		45 (19.1%)	52 (20.2%)	
**Experienced complications during rehabilitation**			< 0.001			0.001
Did not experience	185 (70.9%)	265 (84.4%)		175 (75.8%)	224 (87.5%)	
Experienced	76 (29.1%)	49 (15.6%)		56 (24.2%)	32 (12.5%)	

^1^*n* (%); Median (IQR)

^2^Pearson’s Chi-squared test; Wilcoxon rank sum test; Fisher’s exact test

^a^On stroke admission

^b^On rehabilitation admission

^c^Numbers are not shown due to small number of cases which violates the ethics requirements of reporting

In contrast, patients who achieved FIM efficiency success shared similar characteristics with those who did not, except for a few key differences including fewer number of days delayed in commencing rehabilitation, a shorter duration from the onset of stroke to rehabilitation, a higher FIM total score on admission, and fewer experienced complications during rehabilitation (Table 1).

Among 494 patients who had complete RFG and FIM efficiency data, 211 (42.7%) patients achieved a success in both RFG and FIM efficiency. Conversely, 149 (30.2%) patients were unable to achieve success in either RFG or FIM efficiency. Compared to individuals who did not achieve success in either RFG or FIM efficiency, those who succeeded in both were younger, more likely to be male, had shorter delays in starting rehabilitation, had a shorter time from stroke onset to rehabilitation, achieved higher admission FIM scores, had lower rates of pre-existing cardiac conditions, reported higher rates of pre-stroke employment, and encountered fewer complications during rehabilitation (Table S2). In the group of 298 patients who achieved RFG success, 87 (29.2%) patients didn’t reach FIM efficiency success. Likewise, out of 258 patients with FIM efficiency success, 47 (18.2%) patients didn’t achieve RFG success.

**Fig. 1:**
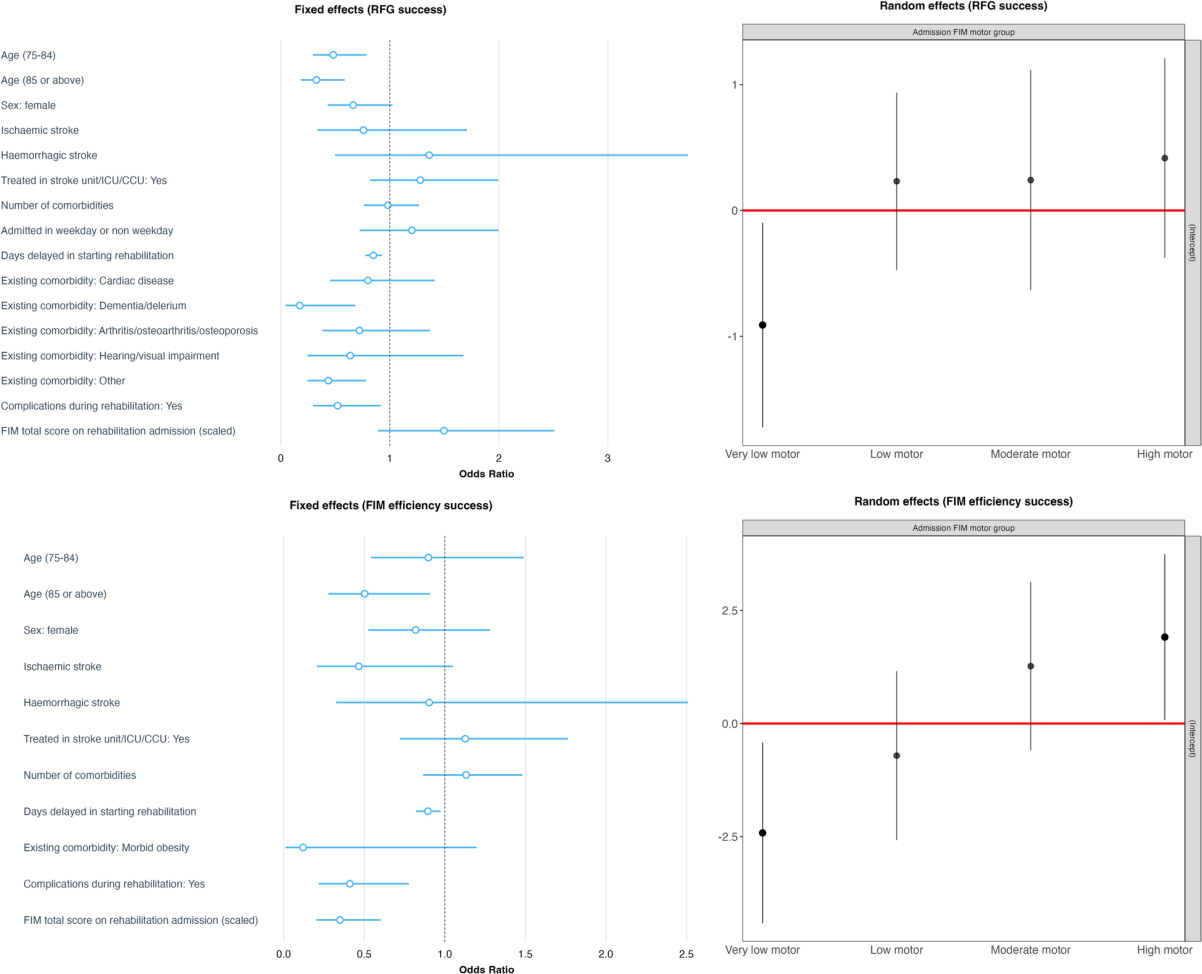
Mixed effects logistical regression for success in Relative Functional Gain and Functional Independence Measure efficiency

In the multivariate regressions (as shown in Fig. 1, supplementary Table S3), number of days delayed in initiation of rehabilitation was associated with decreased likelihood of achieving RFG success [Odds ratio (OR): 0.85, 95% confidence interval (CI): 0.78 to 0.93, *P* < 0.001]. Patients with dementia (OR: 0.17, 95% CI: 0.04 to 0.68, *P* = 0.012) or those who experienced complications during rehabilitation (OR: 0.52, 95% CI: 0.30 to 0.92, *P* = 0.024) were also less likely to achieve RFG success. Compared to patients under 75 years old, those aged between 75 and 84 (OR: 0.48, 95% CI: 0.29 to 0.79, *P* = 0.004), and those aged 85 or above (OR: 0.33, 95% CI: 0.18 to 0.59, *P* < 0.001) were significantly less likely to achieve RFG success.

The significant predictors for not achieving success in FIM efficiency included age ≥ 85 (OR: 0.50, 95% CI: 0.28 to 0.91, *P* = 0.022), number of days delayed in starting rehabilitation (OR: 0.89, 95% CI: 0.82 to 0.97, *P* = 0.010), and experiencing complications during rehabilitation (OR: 0.41, 95% CI: 0.22 to 0.78, *P* = 0.006). A higher FIM total score upon rehabilitation admission (OR: 0.35, 95% CI: 0.20 to 0.60, *P* < 0.001) was associated with a decreased odds to achieve success in FIM efficiency (Fig. 1, Table S4).

As indicated in Table 2, the average daily rehabilitation payment among patients who achieved RFG success was comparable to those who did not. However, the average daily payment for patients who achieved FIM efficiency success ($AU1,516) was significantly higher than the amount paid for patients who did not achieve success ($AU1,075, *P* < 0.001).

**Table 2:** Rehabilitation payment based on AN-SNAP V5 by success in Relative Functional Gain and Functional Independence Measure efficiency

	**Relative functional gain success (> = 0.5)**			**Functional Independence Measure efficiency success (> = 1)**		
**Variable**	**No**, *N* = 196^1^	**Yes**, *N* = 298^1^	***p*-value**^2^	**No**, *N* = 236^1^	**Yes**, *N* = 258^1^	***p*-value**^2^
**Average daily rehabilitation payment**	$AU1,214 (1,050.8, 1,707.5)	$AU1,268 (1,039.6, 1,787.0)	0.816	$AU1,075 (984.5, 1,271.9)	$AU1,516 (1,207.2, 2,030.9)	< 0.001
**Total rehabilitation payment**	29,527 (23,662.2, 41,817.1)	25,352 (18,821.6, 37,950.9)	0.001	32,969 (21,330.5, 53,441.1)	25,352 (18,821.6, 33,816.6)	< 0.001
**Funding model payment categories for rehabilitation**			0.383			< 0.001
Higher outlier	48 (24.5%)	62 (20.8%)		95 (40.3%)	15 (5.8%)	
Inlier	121 (61.7%)	202 (67.8%)		125 (53.0%)	198 (76.7%)	
Low outlier	27 (13.8%)	34 (11.4%)		16 (6.8%)	45 (17.4%)	

^1^Median (IQR); *n* (%)

^2^Wilcoxon rank sum test; Pearson’s Chi-squared test

The results from the multivariate robust regression analysis also revealed the significant positive association between the success in FIM efficiency and the logarithm of the average daily rehabilitation payment (Beta: 0.25, 95% CI: 0.20 to 0.30, *P* < 0.001) (Table S5). The success in RFG was not significantly associated with the logarithm of the average daily rehabilitation payment (Beta: −0.03, 95% CI: −0.09 to 0.03, *P* = 0.266) (Table S6). Additionally, the presence of mental health issues and dementia were found to be positively associated with the logarithm of the average daily rehabilitation payment, with both associations being statistically significant (*P* < 0.05). In contrast, the occurrence of complications during rehabilitation was negatively associated with the logarithm of the average daily rehabilitation payment (*P* < 0.05). Details of covariates adjusted in regression analysis for the logarithm of the average daily rehabilitation payment could be found in Table S5 and S6.

The total rehabilitation payments are less for patients who achieved either RFG success (*P* = 0.001) or FIM efficiency success (*P* < 0.001), compared to those who did not achieve these successes (Table 2). However, the association between the success in RFG and logarithm of total rehabilitation payment was not found to be significant (Beta: 0.04, 95% CI: −0.02 to 0.10, *P* = 0.232) in multivariate robust regression analysis (Table S7). On the contrast, the success in FIM efficiency was significantly and inversely associated with the logarithm of total rehabilitation payment (Beta: −0.18, 95% CI: −0.24 to −0.13, *P* < 0.001) (Table S8). Similarly, the baseline FIM total score upon rehabilitation admission was negatively associated with logarithm of total rehabilitation payment (*P* < 0.05). Covariates adjusted in regression analysis for the logarithm of the average total rehabilitation payment were displayed in Table S7 and S8.

A greater proportion of patients who did not achieve FIM efficiency success had a length of stay in rehabilitation falling into the high outlier category compared to those who achieved FIM efficiency success (*P* < 0.001). However, the distribution of length of stay categories in rehabilitation was similar for patients with and without RFG success (Table 2).

The results of all univariate regressions are presented in supplementary Table S9-S12.

## Discussion

This study found that senior age, complications during rehabilitation, and delayed initiation of rehabilitation decreased the likelihood of success in both RFG and FIM efficiency. Dementia was an additional risk factor for RFG, while a higher FIM total score upon admission negatively affected FIM efficiency. Additionally, it found that the average daily payments for patients who achieved success in FIM efficiency were significantly higher than for those who did not. However, the total payments for patients who succeeded in FIM efficiency were lower than for those who did not. There were no significant differences in either average daily payments or total payments between patients who achieved success in RFG and those who did not.

In this study, the criterion of 10% improvements in FIM as a measure of success has been reported [18] but was not chosen, as we deemed this to be overly conservative. Instead of measuring absolute changes in FIM, RFG was used to assess the recovery of stroke patients, as this approach avoids the ceiling effects associated with absolute changes in functional levels [37]. Hence, the definitions of RFG success and FIM efficiency success were employed to evaluate whether a patient could achieve 50% of their potential improvement or if the rate of functional recovery could reach a desirable level of one point score change in FIM per day. Studies have indicated that improved RFG is associated with improved long-term outcomes, including enhanced health-related quality of life, greater independence, improved mental health status, and reduced odds of hospital readmission among stroke patients [20]. Consequently, it has been suggested that rehabilitation services focus on assisting their patients in achieving higher RFG, regardless of the time needed to do so [20]. However, the efficient allocation of healthcare funds remains a critical concern that needs to be addressed. Therefore, FIM efficiency is also advocated, as it can quantify the speed of functional recovery, reflecting whether the costly and limited rehabilitation resources have been optimally utilised [38]. Thus, achieving success in both RFG and FIM efficiency would be preferable.

Among all the factors associated with a decreased chance of success in RFG and FIM efficiency, delays in commencing rehabilitation and complications during rehabilitation emerged as potential modifiable risk factors. This aligns with previous research that reported a link between delayed commencement of rehabilitation and smaller improvements in FIM scores, as well as lower FIM efficiency [39, 40]. In this cohort of stroke survivors, delays in rehabilitation were predominantly due to unavailability of rehabilitation beds. Therefore, improved access to rehabilitation beds could potentially reduce these delays, thereby increasing the likelihood of achieving success in RFG and FIM efficiency. Another identified modifiable risk factor was complications during rehabilitation. Stroke patients are susceptible to different complications due to the injury caused by stroke and subsequent disabilities [41]. It has been reported that between 44.0% to 88.9% of stroke survivors undergoing rehabilitation experience complications during inpatient rehabilitation [41–45], a proportion higher than that observed in this study. These differences in proportions may be due to variations in research methodologies, such as patient selection criteria [42]. The finding that complications during rehabilitation were associated with reduced odds of the success in RFG and FIM efficiency is consistent with previous reports indicating that complications can impede functional recovery, prolong hospital stays, and lead to worse clinical outcomes [46]. The incidence rates of different types of complications, and the strategies and suggestions for preventing them have been reported in previous studies [42, 47].

This study showed that the FIM score upon admission does not have a significant association with success in RFG. However, it was found that patients with lower admission FIM scores are more likely to achieve success in FIM efficiency, contradicting a report that used simple correlation matrix to find that FIM efficiency was positively associated with admission FIM score [38]. This discrepancy could be attributed to the use of different statistical methods; with our study employing multivariate mix-effect logistical regression. Similar to other research [38],we also report that patients aged 85 or above are less likely to achieve success in FIM efficiency. Similarly, patients aged 75 and above were less likely to achieve success in RFG compared to those younger than 75 years old, consistent with prior findings [48, 49]. The underlying reasons why younger patients achieve better functional outcomes and recover more quickly following a stroke may include their greater recovery capacity, better neuronal plasticity, and fewer comorbidities [50, 51]. Furthermore, the study found that patients with dementia or delirium are less likely to achieve success in RFG as found in a report that showed that patients with better cognitive status upon admission were more likely to achieve functional improvement, while those with delirium were less likely to do so [52].

This study found that success in FIM efficiency was associated with higher average daily payment and lower total payment under the current activity-based funding system. Patients who achieved success in FIM efficiency were less likely to have extended stays classified as “higher outlier” and more likely to have shorter stays classified as “inlier”. Since the “inlier” category has the highest payment weight, and the total payment within this category remains constant, dividing the total payment by fewer days results in a higher average daily payment for these patients. Consequently, these patients were less likely to incur additional costs associated with ‘higher outlier’ and were more likely to have a smaller total payment. This may suggest that achieving success in FIM efficiency could lead to more efficient operational income for rehabilitation services and conserve resources for stroke rehabilitation. This is an optimal scenario as all stakeholders in the system benefit under the current activity-based funding mechanism, including the payer, the rehabilitation service provider, and the service consumer. This aligns with the objectives of the activity-based funding system, which aims to incentivise hospitals to provide better quality healthcare and use funds more efficiently [23]. However, the lack of significant association between both the average daily payment, total payment, and success in RFG suggests that rehabilitation services may not see economic benefits when some patients require a longer time to achieve success in RFG, crucial for patients’ long-term quality of life post-stroke [20]. There is a larger proportion of patients who succeeded in RFG had longer stays categorised as “higher outlier” in the funding model compared to those who achieved a success in FIM efficiency. This creates a conflict in interest between health services providers and the consumers, potentially leading to premature patient discharge before achieving success in RFG.

In Australia, there are three recommended models for stroke rehabilitation: inpatient rehabilitation services, ambulatory rehabilitation (day hospital, outpatient, and home-based rehabilitation), and telemedicine rehabilitation support [53]. After post-acute stroke treatments, surviving patients first undergo inpatient rehabilitation and are then referred to various ambulatory rehabilitation programs [53]. One potential option for balancing the efficient use of inpatient rehabilitation resources with maximising individual patient functional recovery, especially when success in RFG requires a longer stay, is the Early Supported Discharge Community Rehabilitation Program (ESDCRP). This program enables early discharge by providing home-based rehabilitation as a substitute for inpatient care [54]. A meta-analysis found that ESDCRP can reduce hospital stays by approximately seven days, lower the risk of long-term dependency, and decrease the likelihood of institutionalization compared to traditional care, with the most significant benefits observed in stroke survivors with mild-to-moderate disabilities [55]. For patients admitted for ESDCRP, it is recommended that rehabilitation commences within 24 h of hospital discharge and maintains an intensity similar to inpatient rehabilitation [56]. However, providing ESDCRP in rural and remote Australia can be challenging due to long travel distances, low population densities, and shortages of healthcare professionals [57]. Furthermore, the cost-effectiveness of ESDCRP should be evaluated, as the systematic review provides inconclusive evidence regarding whether this program is less costly [55].

### Strengths and limitations

This study utilised data from both hospital and the national rehabilitation database, integrating baseline characteristics at the time of acute stroke admission and upon entry into rehabilitation for analysis. The data linkage with AROC also allowed for the inclusion of patients who were transferred to other facilities for inpatient rehabilitation outside AWH. This study defined rehabilitation success based on RFG and FIM efficiency, considering both the patient’s maximum potential for regaining physical function and the speed of this recovery. The study did not limit its scope to a single type of stroke or initial stroke, thus enhancing the generalisability of the findings to similar patients in the investigated region. The most recent version of National Efficiency Price and AN-SNAP classification was used in the analysis of rehabilitation payment. This provided insights into potential economic implications for hospitals when patients achieved success in RFG and FIM efficiency.

This study has some limitations. The analysis of success in rehabilitation was limited to functional improvements without considering a holistic view, such as quality of life, and psychological aspects. Although the linked data facilitated the inclusion of additional factors in the analysis, this study remains limited by other unknown variables that could have influenced rehabilitation outcomes. Meanwhile, only inpatient rehabilitation was analysed without considering community-based rehabilitation. The amount of rehabilitation payment calculated was a crude amount without adjusting for costly treatments, avoidable readmissions, sentinel incidents, and hospital-acquired complications due to lack of access of relevant data. A single regional hospital was included, which limits the representativeness of this study sample and the overarching study’s generalisability.

## Conclusion

Patients who experienced a delay in initiating rehabilitation, those of advanced age, and those who encountered complications during rehabilitation are less likely to achieve RFG ≥ 0.5 and FIM efficiency ≥ 1. Attaining success in FIM efficiency can enhance the operational income efficiency for rehabilitation services and preserve resources for stroke rehabilitation under the existing activity-based funding model. Nonetheless, if patients take an extended period to attain success in RFG, the rehabilitation services might not witness financial advantages. The insights gained from this study could potentially enhance stroke rehabilitation planning and improve rehabilitation services’ comprehension of the potential impact of success in patient’s functional performance on the payments received.

## Supplementary Information

Supplementary Material 1.

## Data Availability

The data produced or analysed during this study are included in the published article and its supplementary materials. Original individual-level data supporting the findings of this study are available from Albury Wodonga Health and the Australasian Rehabilitation Outcomes Centre, subject to approvals from these institutions.
