# Supplementary material for: Rehabilitation success and related costs following stroke in a regional hospital: a retrospective analysis based on the Australian National Subacute and Non-Acute Patient (AN-SNAP) classification
Source: BMC Health Serv Res. 2025 Jan 23;25:126. doi: 10.1186/s12913-024-12090-w (PMC11755861; doi:10.1186/s12913-024-12090-w)
Supplement: Supplementary file 1 — Supplementary Material 1. [file 12913_2024_12090_MOESM1_ESM.docx]

| **Table S1. Comparison between included and excluded patients** |  |  |  |
| --- | --- | --- | --- |
| **Variable** | **Excluded**, N = 25*^1^* | **Included**, N = 582*^1^* | **p-value***^2^* |
| **Age (years)*** | 74 (65.0, 79.0) | 76 (67.0, 83.0) | 0.150 |
| **Sex*#** |  |  | 0.027 |
| Male | --- | 323 (55.5%) |  |
| Female | --- | 259 (44.5%) |  |
| **Type of stroke*#** |  |  | 0.786 |
| Stroke of unspecified type | --- | 41 (7.0%) |  |
| Ischaemic stroke | --- | 472 (81.1%) |  |
| Haemorrhagic stroke | --- | 69 (11.9%) |  |
| **NIHSS group*#** |  |  | 0.815 |
| Mild | --- | 45 (7.7%) |  |
| moderate | --- | 53 (9.1%) |  |
| severe | --- | 15 (2.6%) |  |
| Unknown | --- | 469 (80.6%) |  |
| *^1^*Median (IQR); n (%) | | | |
| *^2^*Wilcoxon rank sum test; Pearson's Chi-squared test; Fisher's exact test | | | |
| *On stroke admission  #Numbers are not shown due to small number of cases which violates the ethics requirements of reporting. | | | |

| **Table S2: Patient's characteristics by success or failure in both Relative Functional Gain and FIM efficiency** | | | |
| --- | --- | --- | --- |
| **Variable** | Failure in both, N = 149*^1^* | Success in both, N = 211*^1^* | **p-value***^2^* |
| **Age group (years)*** |  |  | 0.000 |
| Under 75 | 45 (30.2%) | 108 (51.2%) |  |
| 75-84 | 56 (37.6%) | 68 (32.2%) |  |
| 85 or more | 48 (32.2%) | 35 (16.6%) |  |
| **Sex*** |  |  | 0.000 |
| Male | 64 (43.0%) | 132 (62.6%) |  |
| Female | 85 (57.0%) | 79 (37.4%) |  |
| **Indigenous status*** |  |  | 1.000 |
| Indigenous | --- | --- |  |
| Not Indigenous | --- | --- |  |
| **Modified Monash Model remoteness*** |  |  | 0.174 |
| metropolitan area/regional centre | 97 (66.0%) | 123 (58.3%) |  |
| Rural area | 50 (34.0%) | 88 (41.7%) |  |
| **Country of birth*** |  |  | 0.422 |
| Australia | 74 (49.7%) | 92 (43.6%) |  |
| Foreign countries | 12 (8.1%) | 15 (7.1%) |  |
| Unknown | 63 (42.3%) | 104 (49.3%) |  |
| **Type of stroke*** |  |  | 0.168 |
| Stroke of unspecified type | 11 (7.4%) | 19 (9.0%) |  |
| Ischaemic stroke | 124 (83.2%) | 159 (75.4%) |  |
| Haemorrhagic stroke | 14 (9.4%) | 33 (15.6%) |  |
| **Treated in a stroke unit or ICU or CCU*** |  |  | 1.000 |
| No | 90 (60.4%) | 128 (60.7%) |  |
| Yes | 59 (39.6%) | 83 (39.3%) |  |
| **Number of comorbidities*** | 2 (1.0, 3.0) | 2 (1.0, 2.0) | 0.128 |
| **Admitted in weekday or non weekday*** |  |  | 0.995 |
| Business day | 116 (77.9%) | 163 (77.3%) |  |
| Weekend/Holiday | 33 (22.1%) | 48 (22.7%) |  |
| **Days delayed in starting rehabilitation^** | 1 (0.0, 3.0) | 1 (0.0, 1.0) | 0.002 |
| **From onset of stroke to rehabilitation (days)** | 11 (7.0, 17.8) | 7 (5.0, 10.0) | 0.000 |
| **FIM total score^** | 66 (39.0, 90.0) | 84 (67.0, 96.0) | 0.000 |
| **Admission FIM motor group^** |  |  | 0.000 |
| Very low motor | 34 (22.8%) | 5 (2.4%) |  |
| Low motor | 27 (18.1%) | 21 (10.0%) |  |
| Moderate motor | 18 (12.1%) | 40 (19.0%) |  |
| High motor | 70 (47.0%) | 145 (68.7%) |  |
| **Rehabilitation services provider^** |  |  | 0.318 |
| Public | 135 (90.6%) | 183 (86.7%) |  |
| Private | 14 (9.4%) | 28 (13.3%) |  |
| **Insurance status^** |  |  | 0.473 |
| Public patient | 68 (45.6%) | 97 (46.0%) |  |
| Private health insurance | 57 (38.3%) | 89 (42.2%) |  |
| Others | 24 (16.1%) | 25 (11.8%) |  |
| **Existing comorbidity: Cardiac disease^** | 40 (26.8%) | 33 (15.6%) | 0.013 |
| **Existing comorbidity: Respiratory disease^** | 11 (7.4%) | 11 (5.2%) | 0.533 |
| **Existing comorbidity: Drug and Alcohol abuse^** | --- | --- | 1.000 |
| **Existing comorbidity: Dementia/delirium^** | --- | --- | 0.375 |
| **Existing comorbidity: Mental health problem^** | 12 (8.1%) | 18 (8.5%) | 1.000 |
| **Existing comorbidity: Hearing/visual impairment^** | 8 (5.4%) | 7 (3.3%) | 0.489 |
| **Existing comorbidity: Diabetes mellites^** | 17 (11.4%) | 22 (10.4%) | 0.902 |
| **Existing comorbidity: Morbid obesity^** | --- | --- | 0.086 |
| **Existing comorbidity: Arthritis/osteoarthritis/osteoporosis^** | 27 (18.1%) | 23 (10.9%) | 0.072 |
| **Existing comorbidity: Chronic pain^** | --- | --- | 0.124 |
| **Existing comorbidity: Cancer^** | 8 (5.4%) | 6 (2.8%) | 0.345 |
| **Existing comorbidity: Renal failure^** | --- | --- | 1.000 |
| **Existing comorbidity: Other^** | 29 (19.5%) | 30 (14.2%) | 0.238 |
| **Need for a carer prior to stroke** |  |  | 0.186 |
| Having carer | 35 (26.3%) | 38 (18.0%) |  |
| No carer | 78 (58.6%) | 138 (65.4%) |  |
| Unknown | 20 (15.0%) | 35 (16.6%) |  |
| **Need for any services prior to stroke** |  |  | 0.053 |
| Yes | 20 (15.0%) | 35 (16.6%) |  |
| No | 78 (58.6%) | 143 (67.8%) |  |
| Unknown | 35 (26.3%) | 33 (15.6%) |  |
| **Employment status prior to stroke** |  |  | 0.004 |
| Retired | 106 (71.1%) | 121 (57.3%) |  |
| Employed | 13 (8.7%) | 44 (20.9%) |  |
| Not employed | 30 (20.1%) | 46 (21.8%) |  |
| **Experienced complications during rehabilitation** |  |  | 0.000 |
| Did not experience | 108 (75.0%) | 187 (89.0%) |  |
| Experienced | 36 (25.0%) | 23 (11.0%) |  |
| *^1^*n (%); Median (IQR) | | | |
| *^2^*Pearson's Chi-squared test; Fisher's exact test; Wilcoxon rank sum test | | | |
| *On stroke admission | | | |
| ^On rehabilitation admission  #Numbers are not shown due to small number of cases which violates the ethics requirements of reporting. | | | |

| **Table S3. Multivariate mixed effects logistical regression for RFG success** | | | |
| --- | --- | --- | --- |
| **Characteristic** | **OR***^1^* | **95% CI***^1^* | **p-value** |
| **Age group (years)*** |  |  |  |
| Under 75 | — | — |  |
| 75-84 | 0.48 | 0.29, 0.79 | 0.004 |
| 85 or more | 0.33 | 0.18, 0.59 | <0.001 |
| **Sex*** |  |  |  |
| Male | — | — |  |
| Female | 0.66 | 0.43, 1.02 | 0.064 |
| **Type of stroke*** |  |  |  |
| Stroke of unspecified type | — | — |  |
| Ischaemic stroke | 0.76 | 0.34, 1.71 | 0.504 |
| Haemorrhagic stroke | 1.36 | 0.50, 3.73 | 0.550 |
| **Treated in a stroke unit or ICU or CCU*** |  |  |  |
| No | — | — |  |
| Yes | 1.28 | 0.82, 1.99 | 0.278 |
| **Number of comorbidities*** | 0.98 | 0.76, 1.27 | 0.890 |
| **Admitted on weekday or non weekday*** |  |  |  |
| Business day | — | — |  |
| Weekend Holiday | 1.20 | 0.72, 2.00 | 0.479 |
| **Days delayed in starting rehabilitation^** | 0.85 | 0.78, 0.93 | <0.001 |
| **Existing comorbidity: Cardiac disease^** |  |  |  |
| No | — | — |  |
| Yes | 0.80 | 0.45, 1.41 | 0.439 |
| **Existing comorbidity: Dementia/delirium^** |  |  |  |
| No | — | — |  |
| Yes | 0.17 | 0.04, 0.68 | 0.012 |
| **Existing comorbidity: Arthritis/osteoarthritis/osteoporosis^** |  |  |  |
| No | — | — |  |
| Yes | 0.72 | 0.38, 1.37 | 0.317 |
| **Existing comorbidity: Hearing/visual impairment^** |  |  |  |
| No | — | — |  |
| Yes | 0.64 | 0.24, 1.67 | 0.360 |
| **Existing comorbidity: Other^** |  |  |  |
| No | — | — |  |
| Yes | 0.44 | 0.24, 0.78 | 0.006 |
| **Experienced complications during rehabilitation** |  |  |  |
| No | — | — |  |
| Yes | 0.52 | 0.30, 0.92 | 0.024 |
| **FIM total score on rehabilitation admission (scaled)^** | 1.50 | 0.89, 2.51 | 0.127 |
| *^1^*OR = Odds Ratio, CI = Confidence Interval | | | |
| **On stroke admission* | | | |
| *^On rehabilitation admission* | | | |

| **Table S4. Multivariate mixed effects logistical regression for FIM efficiency success** | | | |
| --- | --- | --- | --- |
| **Characteristic** | **OR***^1^* | **95% CI***^1^* | **p-value** |
| **Age group (years)*** |  |  |  |
| Under 75 | — | — |  |
| 75-84 | 0.90 | 0.54, 1.49 | 0.677 |
| 85 or more | 0.50 | 0.28, 0.91 | 0.022 |
| **Sex*** |  |  |  |
| Male | — | — |  |
| Female | 0.82 | 0.52, 1.28 | 0.380 |
| **Type of stroke*** |  |  |  |
| Stroke of unspecified type | — | — |  |
| Ischaemic stroke | 0.47 | 0.21, 1.05 | 0.066 |
| Haemorrhagic stroke | 0.90 | 0.33, 2.51 | 0.845 |
| **Treated in a stroke unit or ICU or CCU*** |  |  |  |
| No | — | — |  |
| Yes | 1.13 | 0.72, 1.76 | 0.600 |
| **Number of comorbidities*** | 1.13 | 0.87, 1.48 | 0.364 |
| **Days delayed in starting rehabilitation^** | 0.89 | 0.82, 0.97 | 0.010 |
| **Existing comorbidity: Morbid obesity^** |  |  |  |
| No | — | — |  |
| Yes | 0.12 | 0.01, 1.20 | 0.071 |
| **Experienced complications during rehabilitation** |  |  |  |
| No | — | — |  |
| Yes | 0.41 | 0.22, 0.78 | 0.006 |
| **FIM total score on rehabilitation admission (scaled)^** | 0.35 | 0.20, 0.60 | <0.001 |
| *^1^*OR = Odds Ratio, CI = Confidence Interval | | | |
| *On stroke admission | | | |
| ^On rehabilitation admission | | | |

| **Table S5. Robust regression for average daily rehabilitation payment (FIM efficiency success)** | | | |
| --- | --- | --- | --- |
| **Characteristic** | **Beta** | **95% CI***^1^* | **p-value** |
| **Age group (years)*** |  |  |  |
| Under 75 | — | — |  |
| 75-84 | -0.02 | -0.08, 0.04 | 0.448 |
| 85 or more | -0.03 | -0.09, 0.04 | 0.433 |
| **Sex*** |  |  |  |
| Male | — | — |  |
| Female | 0.02 | -0.03, 0.07 | 0.435 |
| **Type of stroke*** |  |  |  |
| Haemorrhagic stroke | — | — |  |
| Ischaemic stroke | 0.00 | -0.08, 0.08 | 0.964 |
| Stroke of unspecified type | 0.05 | -0.06, 0.16 | 0.393 |
| **NIHSS group*** |  |  |  |
| mild | — | — |  |
| moderate | -0.01 | -0.12, 0.11 | 0.930 |
| severe | -0.02 | -0.21, 0.17 | 0.837 |
| Unknown | -0.07 | -0.16, 0.01 | 0.094 |
| **Number of comorbidities*** | 0.01 | -0.02, 0.04 | 0.591 |
| **Existing comorbidity: Cardiac disease^** |  |  |  |
| No | — | — |  |
| Yes | -0.04 | -0.10, 0.03 | 0.254 |
| **Existing comorbidity: Mental health problem^** |  |  |  |
| No | — | — |  |
| Yes | 0.10 | 0.01, 0.19 | 0.036 |
| **Existing comorbidity: Dementia/delirium^** |  |  |  |
| No | — | — |  |
| Yes | 0.15 | 0.03, 0.28 | 0.018 |
| **Experienced complications during rehabilitation** |  |  |  |
| No | — | — |  |
| Unknown | 0.07 | -0.14, 0.28 | 0.516 |
| Yes | -0.07 | -0.14, -0.01 | 0.030 |
| **FIM total score on rehabilitation admission (scaled)^** | 0.03 | 0.00, 0.06 | 0.022 |
| **FIM efficiency success** |  |  |  |
| No | — | — |  |
| Yes | 0.25 | 0.20, 0.30 | <0.001 |
| *^1^*CI = Confidence Interval | | | |
| *On stroke admission | | | |
| ^On rehabilitation admission | | | |

| **Table S6. Robust regression for average daily rehabilitation payment (RFG success)** | | | |
| --- | --- | --- | --- |
| **Characteristic** | **Beta** | **95% CI***^1^* | **p-value** |
| **Age group (years)*** |  |  |  |
| Under 75 | — | — |  |
| 75-84 | -0.03 | -0.09, 0.03 | 0.353 |
| 85 or more | -0.04 | -0.12, 0.03 | 0.258 |
| **Sex*** |  |  |  |
| Male | — | — |  |
| Female | 0.00 | -0.06, 0.05 | 0.867 |
| **Type of stroke*** |  |  |  |
| Haemorrhagic stroke | — | — |  |
| Ischaemic stroke | -0.04 | -0.12, 0.05 | 0.372 |
| Stroke of unspecified type | 0.05 | -0.08, 0.17 | 0.478 |
| **NIHSS group*** |  |  |  |
| mild | — | — |  |
| moderate | 0.01 | -0.12, 0.13 | 0.915 |
| severe | -0.09 | -0.29, 0.12 | 0.394 |
| Unknown | -0.11 | -0.21, -0.02 | 0.017 |
| **Number of comorbidities*** | 0.01 | -0.02, 0.04 | 0.567 |
| **Existing comorbidity: Cardiac disease^** |  |  |  |
| No | — | — |  |
| Yes | -0.05 | -0.12, 0.02 | 0.126 |
| **Existing comorbidity: Mental health problem^** |  |  |  |
| No | — | — |  |
| Yes | 0.13 | 0.03, 0.23 | 0.012 |
| **Existing comorbidity: Dementia/delirium^** |  |  |  |
| No | — | — |  |
| Yes | 0.19 | 0.05, 0.33 | 0.007 |
| **Experienced complications during rehabilitation** |  |  |  |
| No | — | — |  |
| Unknown | -0.01 | -0.24, 0.22 | 0.954 |
| Yes | -0.12 | -0.19, -0.04 | 0.002 |
| **FIM total score on rehabilitation admission (scaled)^** | 0.05 | 0.02, 0.08 | 0.001 |
| **RFG success** |  |  |  |
| No | — | — |  |
| Yes | -0.03 | -0.09, 0.03 | 0.266 |
| *^1^*CI = Confidence Interval | | | |
| *On stroke admission | | | |
| ^On rehabilitation admission | | | |

| **Table S7. Robust regression for total rehabilitation payment (RFG success)** | | | |
| --- | --- | --- | --- |
| **Characteristic** | **Beta** | **95% CI***^1^* | **p-value** |
| **Age group (years)*** |  |  |  |
| Under 75 | — | — |  |
| 75-84 | -0.08 | -0.15, -0.01 | 0.022 |
| 85 or more | -0.11 | -0.20, -0.03 | 0.006 |
| **Sex*** |  |  |  |
| Male | — | — |  |
| Female | -0.02 | -0.08, 0.04 | 0.529 |
| **Walk independently on admission*** |  |  |  |
| Yes | — | — |  |
| No | -0.10 | -0.21, 0.01 | 0.086 |
| Unknown | -0.04 | -0.15, 0.07 | 0.519 |
| **Number of comorbidities*** | -0.02 | -0.06, 0.01 | 0.153 |
| **Type of stroke*** |  |  |  |
| Stroke of unspecified type | — | — |  |
| Ischaemic stroke | 0.08 | -0.02, 0.18 | 0.125 |
| Haemorrhagic stroke | 0.06 | -0.06, 0.19 | 0.344 |
| **Existing comorbidity: Drug and Alcohol abuse^** |  |  |  |
| No | — | — |  |
| Yes | 0.09 | -0.08, 0.25 | 0.295 |
| **Existing comorbidity: Chronic pain^** |  |  |  |
| No | — | — |  |
| Yes | 0.00 | -0.15, 0.15 | 0.969 |
| **Insurance status^** |  |  |  |
| Public patient | — | — |  |
| Private health insurance | -0.03 | -0.09, 0.03 | 0.281 |
| Others | -0.10 | -0.20, 0.01 | 0.063 |
| **Employment status prior to stroke** |  |  |  |
| Retired | — | — |  |
| Employed | -0.02 | -0.10, 0.06 | 0.615 |
| Not employed | 0.00 | -0.11, 0.10 | 0.961 |
| **Experienced complications during rehabilitation** |  |  |  |
| No | — | — |  |
| Yes | 0.09 | 0.02, 0.17 | 0.017 |
| **Days delayed in starting rehabilitation^** | 0.01 | 0.00, 0.02 | 0.229 |
| **FIM total score on rehabilitation admission (scaled)^** | -0.44 | -0.47, -0.41 | <0.001 |
| **RFG success** |  |  |  |
| No | — | — |  |
| Yes | 0.04 | -0.02, 0.10 | 0.232 |
| *^1^*CI = Confidence Interval | | | |
| *On stroke admission | | | |
| ^On rehabilitation admission | | | |

| **Table S8. Robust regression for total rehabilitation payment (FIM efficiency success**) | | | |
| --- | --- | --- | --- |
| **Characteristic** | **Beta** | **95% CI***^1^* | **p-value** |
| **Age group (years)*** |  |  |  |
| Under 75 | — | — |  |
| 75-84 | -0.07 | -0.14, 0.00 | 0.041 |
| 85 or more | -0.14 | -0.22, -0.06 | <0.001 |
| **Sex*** |  |  |  |
| Male | — | — |  |
| Female | -0.03 | -0.09, 0.02 | 0.244 |
| **Walk independently on admission*** |  |  |  |
| Yes | — | — |  |
| No | -0.09 | -0.19, 0.02 | 0.112 |
| Unknown | -0.02 | -0.13, 0.09 | 0.739 |
| **Number of comorbidities*** | -0.02 | -0.05, 0.01 | 0.232 |
| **Type of stroke*** |  |  |  |
| Stroke of unspecified type | — | — |  |
| Ischaemic stroke | 0.07 | -0.02, 0.17 | 0.130 |
| Haemorrhagic stroke | 0.09 | -0.03, 0.21 | 0.149 |
| **Existing comorbidity: Drug and Alcohol abuse^** |  |  |  |
| No | — | — |  |
| Yes | 0.07 | -0.09, 0.22 | 0.421 |
| **Existing comorbidity: Chronic pain^** |  |  |  |
| No | — | — |  |
| Yes | -0.02 | -0.17, 0.13 | 0.788 |
| **Insurance status^** |  |  |  |
| Public patient | — | — |  |
| Private health insurance | -0.03 | -0.09, 0.03 | 0.284 |
| Others | -0.07 | -0.17, 0.03 | 0.185 |
| **Employment status prior to stroke** |  |  |  |
| Retired | — | — |  |
| Employed | -0.02 | -0.10, 0.07 | 0.711 |
| Not employed | 0.02 | -0.08, 0.12 | 0.634 |
| **Experienced complications during rehabilitation** |  |  |  |
| No | — | — |  |
| Yes | 0.05 | -0.02, 0.13 | 0.155 |
| **Days delayed in starting rehabilitation^** | 0.00 | -0.01, 0.01 | 0.638 |
| **FIM total score on rehabilitation admission (scaled)^** | -0.43 | -0.46, -0.40 | <0.001 |
| **FIM efficiency success** |  |  |  |
| No | — | — |  |
| Yes | -0.18 | -0.24, -0.13 | <0.001 |
| *^1^*CI = Confidence Interval | | | |
| *On stroke admission | | | |
| ^On rehabilitation admission | | | |

| **Table S9. Univariate mixed effects logistical regression for RFG success** | | | |
| --- | --- | --- | --- |
| **Characteristic** | **OR***^1^* | **95% CI***^1^* | **p-value** |
| **Age group (years)*** |  |  |  |
| Under 75 | — | — |  |
| 75-84 | 0.36 | 0.24, 0.54 | <0.001 |
| 85 or more | 0.24 | 0.15, 0.38 | <0.001 |
| **Sex*** |  |  |  |
| Male | — | — |  |
| Female | 0.53 | 0.38, 0.76 | <0.001 |
| **Country of birth*** |  |  |  |
| Australia | — | — |  |
| Foreign countries | 1.46 | 0.66, 3.22 | 0.355 |
| Unknown | 1.32 | 0.92, 1.88 | 0.133 |
| **Social economic Status*** |  |  |  |
| Below median | — | — |  |
| Median and above | 0.95 | 0.67, 1.36 | 0.793 |
| **Walk independently on admission*** |  |  |  |
| Yes | — | — |  |
| No | 1.26 | 0.60, 2.66 | 0.545 |
| Unknown | 1.55 | 0.74, 3.27 | 0.246 |
| **NIHSS group*** |  |  |  |
| mild | — | — |  |
| moderate | 1.56 | 0.66, 3.72 | 0.314 |
| severe | 0.21 | 0.04, 1.13 | 0.069 |
| Unknown | 0.87 | 0.45, 1.66 | 0.664 |
| **Number of comorbidities*** | 0.87 | 0.71, 1.07 | 0.190 |
| **Treated in a stroke unit or ICU or CCU*** |  |  |  |
| No | — | — |  |
| Yes | 1.09 | 0.76, 1.56 | 0.634 |
| **Indigenous status*** |  |  |  |
| No | — | — |  |
| Yes | 0.43 | 0.07, 2.52 | 0.347 |
| **Modified Monash Model remoteness*** |  |  |  |
| metropolitan area/regional centre | — | — |  |
| Rural area | 1.23 | 0.86, 1.75 | 0.260 |
| **Admitted in daytime or night-time*** |  |  |  |
| Daytime | — | — |  |
| Nighttime | 1.15 | 0.81, 1.64 | 0.420 |
| **Admitted in weekday or non weekday*** |  |  |  |
| Business day | — | — |  |
| Weekend or Holiday | 1.46 | 0.96, 2.22 | 0.077 |
| **Type of stroke*** |  |  |  |
| Stroke of unspecified type | — | — |  |
| Ischaemic stroke | 1.10 | 0.57, 2.13 | 0.778 |
| Haemorrhagic stroke | 1.83 | 0.80, 4.19 | 0.153 |
| **Existing comorbidity: Cardiac disease^** |  |  |  |
| No | — | — |  |
| Yes | 0.60 | 0.39, 0.92 | 0.018 |
| **Existing comorbidity: Respiratory disease^** |  |  |  |
| No | — | — |  |
| Yes | 0.75 | 0.39, 1.44 | 0.389 |
| **Existing comorbidity: Drug and Alcohol abuse^** |  |  |  |
| No | — | — |  |
| Yes | 0.50 | 0.17, 1.46 | 0.205 |
| **Existing comorbidity: Mental health problem^** |  |  |  |
| No | — | — |  |
| Yes | 0.70 | 0.37, 1.32 | 0.272 |
| **Existing comorbidity: Diabetes mellites^** |  |  |  |
| No | — | — |  |
| Yes | 0.85 | 0.50, 1.44 | 0.546 |
| **Existing comorbidity: Morbid obesity^** |  |  |  |
| No | — | — |  |
| Yes | 0.92 | 0.29, 2.94 | 0.887 |
| **Existing comorbidity: Chronic pain^** |  |  |  |
| No | — | — |  |
| Yes | 0.68 | 0.22, 2.06 | 0.496 |
| **Existing comorbidity: Cancer^** |  |  |  |
| No | — | — |  |
| Yes | 0.55 | 0.23, 1.29 | 0.168 |
| **Existing comorbidity: Dementia/delirium^** |  |  |  |
| No | — | — |  |
| Yes | 0.16 | 0.05, 0.50 | 0.001 |
| **Existing comorbidity: Renal failure^** |  |  |  |
| No | — | — |  |
| Yes | 1.26 | 0.35, 4.61 | 0.724 |
| **Existing comorbidity: Arthritis/osteoarthritis/osteoporosis^** |  |  |  |
| No | — | — |  |
| Yes | 0.54 | 0.32, 0.91 | 0.021 |
| **Existing comorbidity: Hearing/visual impairment^** |  |  |  |
| No | — | — |  |
| Yes | 0.47 | 0.20, 1.09 | 0.077 |
| **Existing comorbidity: Other^** |  |  |  |
| No | — | — |  |
| Yes | 0.42 | 0.26, 0.67 | <0.001 |
| **From onset of stroke to rehabilitation (days)** | 0.98 | 0.96, 0.99 | 0.005 |
| **Experienced complications during rehabilitation** |  |  |  |
| No | — | — |  |
| Yes | 0.56 | 0.36, 0.86 | 0.009 |
| Unknown | 0.08 | 0.01, 0.68 | 0.021 |
| **Days delayed in starting rehabilitation^** | 0.86 | 0.79, 0.93 | <0.001 |
| **FIM total score on rehabilitation admission (scaled)^** | 2.05 | 1.50, 2.80 | <0.001 |

| *^1^*OR = Odds Ratio, CI = Confidence Interval |
| --- |
| **On stroke admission* |
| *^On rehabilitation admission* |

| **Table S10. Univariate mixed effects logistical regression for FIM efficiency success** | | | | |
| --- | --- | --- | --- | --- |
| **Characteristic** | **N** | **OR***^1^* | **95% CI***^1^* | **p-value** |
| **Age group (years)*** | 494 |  |  |  |
| Under 75 |  | — | — |  |
| 75-84 |  | 1.03 | 0.68, 1.58 | 0.877 |
| 85 or more |  | 0.73 | 0.45, 1.18 | 0.203 |
| **Sex*** | 494 |  |  |  |
| Male |  | — | — |  |
| Female |  | 0.83 | 0.57, 1.20 | 0.313 |
| **Country of birth*** | 494 |  |  |  |
| Australia |  | — | — |  |
| Foreign countries |  | 1.18 | 0.55, 2.57 | 0.670 |
| Unknown |  | 1.07 | 0.73, 1.57 | 0.717 |
| **Social economic Status*** | 492 |  |  |  |
| Below median |  | — | — |  |
| Median and above |  | 0.88 | 0.60, 1.28 | 0.503 |
| **Walk independently on admission*** | 494 |  |  |  |
| Yes |  | — | — |  |
| No |  | 1.13 | 0.51, 2.48 | 0.768 |
| Unknown |  | 1.10 | 0.50, 2.42 | 0.810 |
| **NIHSS group*** | 494 |  |  |  |
| mild |  | — | — |  |
| moderate |  | 0.89 | 0.37, 2.14 | 0.788 |
| severe |  | 0.41 | 0.09, 1.92 | 0.257 |
| Unknown |  | 0.62 | 0.32, 1.21 | 0.161 |
| **Number of comorbidities*** | 494 | 1.02 | 0.81, 1.27 | 0.872 |
| **Treated in a stroke unit or ICU or CCU*** | 494 |  |  |  |
| No |  | — | — |  |
| Yes |  | 1.03 | 0.70, 1.50 | 0.890 |
| **Indigenous status*** | 493 |  |  |  |
| No |  | — | — |  |
| Yes |  | 1.48 | 0.22, 10.2 | 0.689 |
| **Modified Monash Model remoteness*** | 492 |  |  |  |
| metropolitan area/regional centre |  | — | — |  |
| Rural area |  | 1.20 | 0.82, 1.76 | 0.337 |
| **Admitted in daytime or night-time*** | 494 |  |  |  |
| Daytime |  | — | — |  |
| Nighttime |  | 0.84 | 0.58, 1.22 | 0.355 |
| **Admitted in weekday or non weekday*** | 494 |  |  |  |
| Business day |  | — | — |  |
| Weekend or Holiday |  | 0.96 | 0.62, 1.49 | 0.863 |
| **Type of stroke*** | 494 |  |  |  |
| Stroke of unspecified type |  | — | — |  |
| Ischaemic stroke |  | 0.67 | 0.33, 1.36 | 0.266 |
| Haemorrhagic stroke |  | 1.45 | 0.59, 3.56 | 0.413 |
| **Existing comorbidity: Cardiac disease^** | 494 |  |  |  |
| No |  | — | — |  |
| Yes |  | 0.68 | 0.43, 1.08 | 0.100 |
| **Existing comorbidity: Respiratory disease^** | 494 |  |  |  |
| No |  | — | — |  |
| Yes |  | 0.71 | 0.35, 1.45 | 0.351 |
| **Existing comorbidity: Drug and Alcohol abuse^** | 494 |  |  |  |
| No |  | — | — |  |
| Yes |  | 1.61 | 0.48, 5.35 | 0.437 |
| **Existing comorbidity: Mental health problem^** | 494 |  |  |  |
| No |  | — | — |  |
| Yes |  | 1.36 | 0.68, 2.74 | 0.384 |
| **Existing comorbidity: Diabetes mellites^** | 494 |  |  |  |
| No |  | — | — |  |
| Yes |  | 0.91 | 0.50, 1.67 | 0.768 |
| **Existing comorbidity: Morbid obesity^** | 494 |  |  |  |
| No |  | — | — |  |
| Yes |  | 0.10 | 0.01, 0.83 | 0.033 |
| **Existing comorbidity: Chronic pain^** | 494 |  |  |  |
| No |  | — | — |  |
| Yes |  | 0.62 | 0.19, 2.03 | 0.431 |
| **Existing comorbidity: Cancer^** | 494 |  |  |  |
| No |  | — | — |  |
| Yes |  | 0.63 | 0.24, 1.67 | 0.354 |
| **Existing comorbidity: Dementia/delirium^** | 494 |  |  |  |
| No |  | — | — |  |
| Yes |  | 2.31 | 0.82, 6.49 | 0.114 |
| **Existing comorbidity: Renal failure^** | 494 |  |  |  |
| No |  | — | — |  |
| Yes |  | 0.99 | 0.24, 4.05 | 0.991 |
| **Existing comorbidity: Arthritis/osteoarthritis/osteoporosis^** | 494 |  |  |  |
| No |  | — | — |  |
| Yes |  | 1.07 | 0.61, 1.87 | 0.825 |
| **Existing comorbidity: Hearing/visual impairment^** | 494 |  |  |  |
| No |  | — | — |  |
| Yes |  | 0.97 | 0.41, 2.31 | 0.940 |
| **Existing comorbidity: Other^** | 494 |  |  |  |
| No |  | — | — |  |
| Yes |  | 1.00 | 0.60, 1.67 | 0.998 |
| **From onset of stroke to rehabilitation (days)** | 451 | 0.98 | 0.97, 1.00 | 0.052 |
| **Experienced complications during rehabilitation** | 494 |  |  |  |
| No |  | — | — |  |
| Yes |  | 0.49 | 0.30, 0.83 | 0.007 |
| Unknown |  | 0.26 | 0.05, 1.37 | 0.112 |
| **Days delayed in starting rehabilitation^** | 415 | 0.91 | 0.84, 0.98 | 0.017 |
| **FIM total score on rehabilitation admission (scaled)^** | 494 | 0.46 | 0.29, 0.72 | <0.001 |
| *^1^*OR = Odds Ratio, CI = Confidence Interval | | | | |
| **On stroke admission* | | | | |
| *^On rehabilitation admission* | | | | |

| **Table S11. Univariate Robust regression for average daily rehabilitation payment** | | | |
| --- | --- | --- | --- |
| **Characteristic** | **Beta** | **95% CI***^1^* | **p-value** |
| **Age group (years)*** |  |  |  |
| Under 75 | — | — |  |
| 75-84 | -0.02 | -0.08, 0.04 | 0.517 |
| 85 or more | -0.04 | -0.11, 0.03 | 0.266 |
| **Sex*** |  |  |  |
| Male | — | — |  |
| Female | 0.00 | -0.05, 0.05 | 0.942 |
| **Country of birth*** |  |  |  |
| Australia | — | — |  |
| Foreign countries | -0.02 | -0.13, 0.09 | 0.758 |
| Unknown | -0.01 | -0.06, 0.05 | 0.746 |
| **Social economic Status*** |  |  |  |
| Below median | — | — |  |
| Median and above | -0.04 | -0.09, 0.02 | 0.165 |
| **Walk independently on admission*** |  |  |  |
| Yes | — | — |  |
| No | -0.03 | -0.14, 0.08 | 0.613 |
| Unknown | -0.05 | -0.16, 0.07 | 0.415 |
| **NIHSS group*** |  |  |  |
| mild | — | — |  |
| moderate | -0.02 | -0.15, 0.10 | 0.709 |
| severe | -0.19 | -0.39, 0.00 | 0.056 |
| Unknown | -0.12 | -0.22, -0.03 | 0.010 |
| **Number of comorbidities*** | 0.00 | -0.03, 0.03 | 0.802 |
| **Treated in a stroke unit or ICU or CCU*** |  |  |  |
| No | — | — |  |
| Yes | 0.01 | -0.04, 0.06 | 0.675 |
| **Indigenous status*** |  |  |  |
| No | — | — |  |
| Yes | 0.06 | -0.20, 0.32 | 0.659 |
| **Modified Monash Model remoteness*** |  |  |  |
| metropolitan area/regional centre | — | — |  |
| Rural area | 0.02 | -0.04, 0.07 | 0.551 |
| **Admitted in daytime or night-time*** |  |  |  |
| Daytime | — | — |  |
| Nighttime | 0.00 | -0.06, 0.05 | 0.854 |
| **Admitted in weekday or non weekday*** |  |  |  |
| Business day | — | — |  |
| Weekend or Holiday | 0.00 | -0.06, 0.07 | 0.889 |
| **Type of stroke*** |  |  |  |
| Stroke of unspecified type | — | — |  |
| Ischaemic stroke | -0.09 | -0.20, 0.01 | 0.072 |
| Haemorrhagic stroke | -0.07 | -0.20, 0.05 | 0.240 |
| **Existing comorbidity: Cardiac disease^** |  |  |  |
| No | — | — |  |
| Yes | -0.09 | -0.16, -0.03 | 0.006 |
| **Existing comorbidity: Respiratory disease^** |  |  |  |
| No | — | — |  |
| Yes | 0.02 | -0.08, 0.12 | 0.680 |
| **Existing comorbidity: Drug and Alcohol abuse^** |  |  |  |
| No | — | — |  |
| Yes | 0.11 | -0.05, 0.28 | 0.179 |
| **Existing comorbidity: Mental health problem^** |  |  |  |
| No | — | — |  |
| Yes | 0.11 | 0.01, 0.21 | 0.028 |
| **Existing comorbidity: Diabetes mellites^** |  |  |  |
| No | — | — |  |
| Yes | -0.04 | -0.13, 0.04 | 0.317 |
| **Existing comorbidity: Morbid obesity^** |  |  |  |
| No | — | — |  |
| Yes | 0.05 | -0.13, 0.23 | 0.591 |
| **Existing comorbidity: Chronic pain^** |  |  |  |
| No | — | — |  |
| Yes | 0.01 | -0.15, 0.17 | 0.911 |
| **Existing comorbidity: Cancer^** |  |  |  |
| No | — | — |  |
| Yes | -0.06 | -0.20, 0.08 | 0.407 |
| **Existing comorbidity: Dementia/delirium^** |  |  |  |
| No | — | — |  |
| Yes | 0.16 | 0.02, 0.29 | 0.020 |
| **Existing comorbidity: Renal failure^** |  |  |  |
| No | — | — |  |
| Yes | 0.00 | -0.19, 0.19 | 0.998 |
| **Existing comorbidity: Arthritis/osteoarthritis/osteoporosis^** |  |  |  |
| No | — | — |  |
| Yes | -0.03 | -0.11, 0.05 | 0.435 |
| **Existing comorbidity: Hearing/visual impairment^** |  |  |  |
| No | — | — |  |
| Yes | 0.02 | -0.10, 0.15 | 0.704 |
| **Existing comorbidity: Other^** |  |  |  |
| No | — | — |  |
| Yes | -0.01 | -0.08, 0.06 | 0.822 |
| **Experienced complications during rehabilitation** |  |  |  |
| No | — | — |  |
| Unknown | -0.02 | -0.23, 0.20 | 0.873 |
| Yes | -0.14 | -0.20, -0.07 | <0.001 |
| **From onset of stroke to rehabilitation (days)** | 0.00 | 0.00, 0.00 | 0.799 |
| **Days delayed in starting rehabilitation^** | 0.00 | -0.01, 0.01 | 0.390 |
| **FIM total score on rehabilitation admission (scaled)^** | 0.06 | 0.03, 0.08 | <0.001 |
| **Need for a carer prior to stroke** |  |  |  |
| No carer | — | — |  |
| Having carer | 0.01 | -0.06, 0.08 | 0.818 |
| Unknown | -0.03 | -0.10, 0.03 | 0.327 |
| **Need for any services prior to stroke** |  |  |  |
| No | — | — |  |
| Yes | -0.05 | -0.11, 0.02 | 0.180 |
| Unknown | -0.05 | -0.12, 0.02 | 0.199 |
| **Insurance status^** |  |  |  |
| Public patient | — | — |  |
| Private health insurance | -0.01 | -0.07, 0.05 | 0.716 |
| Others | -0.01 | -0.10, 0.07 | 0.775 |
| **Employment status prior to stroke** |  |  |  |
| Retired | — | — |  |
| Employed | 0.06 | -0.01, 0.13 | 0.111 |
| Not employed | -0.05 | -0.11, 0.02 | 0.180 |
| **FIM efficiency success** |  |  |  |
| No | — | — |  |
| Yes | 0.29 | 0.24, 0.34 | <0.001 |
| **RFG success** |  |  |  |
| No | — | — |  |
| Yes | 0.01 | -0.04, 0.06 | 0.727 |

| *^1^*OR = Odds Ratio, CI = Confidence Interval |
| --- |
| **On stroke admission* |
| *^On rehabilitation admission* |

| **Table S12. Univariate Robust regression for total rehabilitation payment** | | | |
| --- | --- | --- | --- |
| **Characteristic** | **Beta** | **95% CI***^1^* | **p-value** |
| **Age group (years)*** |  |  |  |
| Under 75 | — | — |  |
| 75-84 | -0.05 | -0.15, 0.06 | 0.370 |
| 85 or more | -0.05 | -0.17, 0.07 | 0.393 |
| **Sex*** |  |  |  |
| Male | — | — |  |
| Female | 0.05 | -0.04, 0.15 | 0.253 |
| **Country of birth*** |  |  |  |
| Australia | — | — |  |
| Foreign countries | 0.07 | -0.12, 0.26 | 0.466 |
| Unknown | 0.04 | -0.06, 0.13 | 0.451 |
| **Social economic Status*** |  |  |  |
| Below median | — | — |  |
| Median and above | 0.03 | -0.07, 0.12 | 0.570 |
| **Walk independently on admission*** |  |  |  |
| Yes | — | — |  |
| No | 0.24 | 0.04, 0.43 | 0.019 |
| Unknown | 0.25 | 0.06, 0.45 | 0.012 |
| **NIHSS group*** |  |  |  |
| mild | — | — |  |
| moderate | 0.12 | -0.09, 0.32 | 0.276 |
| severe | 0.55 | 0.21, 0.88 | 0.001 |
| Unknown | 0.18 | 0.02, 0.33 | 0.029 |
| **Number of comorbidities*** | 0.11 | 0.06, 0.17 | <0.001 |
| **Treated in a stroke unit or ICU or CCU*** |  |  |  |
| No | — | — |  |
| Yes | 0.05 | -0.04, 0.15 | 0.269 |
| **Indigenous status*** |  |  |  |
| No | — | — |  |
| Yes | 0.36 | -0.09, 0.82 | 0.117 |
| **Modified Monash Model remoteness*** |  |  |  |
| metropolitan area/regional centre | — | — |  |
| Rural area | -0.07 | -0.16, 0.03 | 0.173 |
| **Admitted in daytime or night-time*** |  |  |  |
| Daytime | — | — |  |
| Nighttime | 0.03 | -0.06, 0.13 | 0.467 |
| **Admitted in weekday or non weekday*** |  |  |  |
| Business day | — | — |  |
| Weekend or Holiday | 0.07 | -0.03, 0.18 | 0.176 |
| **Type of stroke*** |  |  |  |
| Stroke of unspecified type | — | — |  |
| Ischaemic stroke | 0.22 | 0.04, 0.39 | 0.014 |
| Haemorrhagic stroke | 0.31 | 0.10, 0.52 | 0.005 |
| **Existing comorbidity: Cardiac disease^** |  |  |  |
| No | — | — |  |
| Yes | 0.03 | -0.08, 0.15 | 0.575 |
| **Existing comorbidity: Respiratory disease^** |  |  |  |
| No | — | — |  |
| Yes | 0.04 | -0.14, 0.21 | 0.692 |
| **Existing comorbidity: Drug and Alcohol abuse^** |  |  |  |
| No | — | — |  |
| Yes | 0.26 | -0.02, 0.55 | 0.068 |
| **Existing comorbidity: Mental health problem^** |  |  |  |
| No | — | — |  |
| Yes | 0.06 | -0.11, 0.23 | 0.501 |
| **Existing comorbidity: Diabetes mellites^** |  |  |  |
| No | — | — |  |
| Yes | 0.15 | 0.00, 0.30 | 0.044 |
| **Existing comorbidity: Morbid obesity^** |  |  |  |
| No | — | — |  |
| Yes | 0.15 | -0.17, 0.47 | 0.355 |
| **Existing comorbidity: Chronic pain^** |  |  |  |
| No | — | — |  |
| Yes | 0.33 | 0.06, 0.60 | 0.019 |
| **Existing comorbidity: Cancer^** |  |  |  |
| No | — | — |  |
| Yes | -0.05 | -0.29, 0.20 | 0.703 |
| **Existing comorbidity: Dementia/delirium^** |  |  |  |
| No | — | — |  |
| Yes | -0.07 | -0.30, 0.16 | 0.547 |
| **Existing comorbidity: Renal failure^** |  |  |  |
| No | — | — |  |
| Yes | 0.20 | -0.14, 0.54 | 0.238 |
| **Existing comorbidity: Arthritis/osteoarthritis/osteoporosis^** |  |  |  |
| No | — | — |  |
| Yes | 0.09 | -0.05, 0.22 | 0.220 |
| **Existing comorbidity: Hearing/visual impairment^** |  |  |  |
| No | — | — |  |
| Yes | -0.11 | -0.33, 0.10 | 0.310 |
| **Existing comorbidity: Other^** |  |  |  |
| No | — | — |  |
| Yes | -0.05 | -0.18, 0.08 | 0.425 |
| **Experienced complications during rehabilitation** |  |  |  |
| No | — | — |  |
| Unknown | -0.22 | -0.60, 0.17 | 0.266 |
| Yes | 0.41 | 0.29, 0.53 | <0.001 |
| **From onset of stroke to rehabilitation (days)** | 0.01 | 0.01, 0.01 | <0.001 |
| **Days delayed in starting rehabilitation^** | 0.02 | 0.01, 0.04 | 0.007 |
| **FIM total score on rehabilitation admission (scaled)^** | -0.44 | -0.46, -0.41 | <0.001 |
| **Rehabilitation services provider^** |  |  |  |
| Public | — | — |  |
| Private | -0.29 | -0.45, -0.12 | <0.001 |
| **Need for a carer prior to stroke** |  |  |  |
| No carer | — | — |  |
| Having carer | -0.07 | -0.19, 0.05 | 0.236 |
| Unknown | 0.04 | -0.07, 0.16 | 0.472 |
| **Need for any services prior to stroke** |  |  |  |
| No | — | — |  |
| Yes | 0.07 | -0.04, 0.19 | 0.212 |
| Unknown | 0.05 | -0.07, 0.17 | 0.373 |
| **Insurance status^** |  |  |  |
| Public patient | — | — |  |
| Private health insurance | -0.11 | -0.21, 0.00 | 0.041 |
| Others | -0.07 | -0.22, 0.09 | 0.401 |
| **Employment status prior to stroke** |  |  |  |
| Retired | — | — |  |
| Employed | -0.16 | -0.28, -0.03 | 0.013 |
| Not employed | 0.02 | -0.10, 0.14 | 0.760 |
| **FIM efficiency success** |  |  |  |
| No | — | — |  |
| Yes | -0.28 | -0.37, -0.20 | <0.001 |
| **RFG success** |  |  |  |
| No | — | — |  |
| Yes | -0.15 | -0.25, -0.06 | 0.002 |

| *^1^*OR = Odds Ratio, CI = Confidence Interval |
| --- |
| **On stroke admission* |
| *^On rehabilitation admission* |
